# Disruptions of Circadian Rhythms and Thrombolytic Therapy During Ischemic Stroke Intervention

**DOI:** 10.3389/fnins.2021.675732

**Published:** 2021-06-10

**Authors:** Jennifer A. Liu, James C. Walton, A. Courtney DeVries, Randy J. Nelson

**Affiliations:** ^1^Department of Neuroscience, Rockefeller Neuroscience Institute, West Virginia University, Morgantown, WV, United States; ^2^Department of Medicine, Division of Oncology/Hematology, West Virginia University, Morgantown, WV, United States; ^3^West Virginia University Cancer Institute, West Virginia University, Morgantown, WV, United States

**Keywords:** circadian rhythm, stroke, prothrombotic state, TPA, hemorrhagic transformation, light at night (LAN), time of day, thrombolysis (tPA)

## Abstract

Several endogenous and exogenous factors interact to influence stroke occurrence, in turn contributing to discernable daily distribution patterns in the frequency and severity of cerebrovascular events. Specifically, strokes that occur during the morning tend to be more severe and are associated with elevated diastolic blood pressure, increased hospital stay, and worse outcomes, including mortality, compared to strokes that occur later in the day. Furthermore, disrupted circadian rhythms are linked to higher risk for stroke and play a role in stroke outcome. In this review, we discuss the interrelation among core clock genes and several factors contributing to ischemic outcomes, sources of disrupted circadian rhythms, the implications of disrupted circadian rhythms in foundational stroke scientific literature, followed by a review of clinical implications. In addition to highlighting the distinct daily pattern of onset, several aspects of physiology including immune response, endothelial/vascular and blood brain barrier function, and fibrinolysis are under circadian clock regulation; disrupted core clock gene expression patterns can adversely affect these physiological processes, leading to a prothrombotic state. Lastly, we discuss how the timing of ischemic onset increases morning resistance to thrombolytic therapy and the risk of hemorrhagic transformation.

## Introduction

Stroke, caused by a sudden interruption to the blood supply of the brain, has a wide range of detrimental consequences that have contributed to its recognition as the leading cause of disability and second most common cause of death worldwide. It has serious social and economic consequences for both the individual and society. Ischemic strokes account for the majority of all strokes (87%), with intracerebral hemorrhages and subarachnoid hemorrhages being far less common (10% and 3%, respectively (Mozaffarian et al., 2016). Current FDA approved pharmaceutical treatment for acute ischemia is limited to thrombolytic intervention, which functions to restore blood flow, yet patients often still suffer long term motor, behavioral, and cognitive deficits due to a lack of interventions focused on promoting neuronal post-stroke recovery (Stubblefield and Lechleiter, 2019). Tissue plasminogen activator (tPA), is a naturally produced protein in endothelial cells that functions as a serine protease, catalyzing the conversion of plasminogen to plasmin, which acts as a key player in fibrinolysis. This protease has been used as a rapid and effective treatment during ischemic strokes by initiating reperfusion of the affected brain region, however, the therapeutic efficacy is limited to a 3–4.5-h time window from stroke onset (Cheng and Kim, 2015). Due to this limited intervention time, only about 3–5% of patients are eligible to receive tPA treatment (Fonarow et al., 2011) and 2–8% of that eligible population experience intracerebral hemorrhage as a consequence of receiving tPA (Miller et al., 2011).

Several aspects of physiology and behavior that are involved and contribute to ischemic injury are governed by the circadian system. Endogenous biological rhythms, modulated through external cues such as light, are important for adapting to predictable changes in the environment and maintaining internal physiology, which suggests the importance of time of day on health (Lowrey and Takahashi, 2000; Golombek and Rosenstein, 2010; Patke et al., 2020). Disruptions to these biological rhythms can dysregulate physiological function, including the cardiovascular system, metabolic and endocrine function and aspects of immune function (Davidson et al., 2010; Chen and Yang, 2015; Bedrosian et al., 2016; Serin and Acar Tek, 2019). In the context of ischemic stroke, several risk factors, including those described above, contribute to the severity of damage, patient recovery, and response to thrombolytic treatment (Seet et al., 2014; Arboix, 2015). Variability of patient response to ischemic stroke and its treatments, as well as considerations for the lack of consistency, suggests a need to consider circadian rhythms in order to optimize therapeutic strategies. This review will discuss the circadian regulation of several exogenous factors that influence risk for stroke and highlight time of day alterations in the efficacy of tPA administration and risk for hemorrhagic transformation.

## Circadian Rhythms, the Mammalian Clock

Circadian rhythms are endogenous daily fluctuations in physiology and behavior driven through biological oscillators or clocks, and have been documented in most organisms, from bacteria to vertebrates (Bhadra et al., 2017). Physiological systems are optimized to promote biological adaptations and survival by adapting to the 24-h solar day (Daan and Aschoff, 1982). Circadian rhythms are driven and regulated through a transcriptional-translational feedback loop of core clock genes lasting approximately 24 h and are synchronized through exposure to the external light-dark cycle as a result of Earth’s daily rotation. These external factors that can influence and synchronize the circadian rhythms to the environment are known as zeitgebers (German word for “time-giver”). Zeitgeber time (ZT) is often used in circadian research to standardize time of day, and refers to the unit time based on the duration of time from initiation of the zeitgeber; for example, the onset of lights would be designated ZT 0, whereas the onset of darkness 12 h later would be designated ZT 12. Thus, diurnal animals would be most active between ZT 0 and ZT12, whereas nocturnal organisms would be most active between ZT 12 and ZT 24 (Aschoff, 1981). Another fundamental component of circadian rhythms is the ability to persist in the absence of any external or environmental cues. Other external cues, including feeding, physical activity, and social cues, have the capacity to entrain circadian rhythms as well, but generally less effectively as light (Aschoff et al., 1971; Stephan, 2002; Lewis et al., 2018).

### Mechanism of the Molecular Circadian Clock

The mechanism underlying cellular circadian clocks arises from an a transcriptional-translational autoregulatory feedback loop from a distinct set of genes including the Circadian Locomotor Output Cycles Kaput (*Clock*), Brain and Muscle ARNT-like Protein 1 (*Bmal1*), Period (*Per*), and Cytochrome (*Cry*). *Clock* and *Bmal1* encode for proteins containing bHlh-PAS domains, which form the positive arm of the feedback circuit by heterodimerizing to initiate transcription through binding to E-boxes (5′-CACGTG-3′) and (5′-CACGTT-3′) in the promoter of target genes. In turn, Per and Cry dimerize, acting as the negative limb of the feedback loop to inhibit *Clock:Bmal1* transcriptional activity for the cycle to repeat, and degradation of Per and Cry proteins are responsible for terminating repression and restarting transcription. A second transcriptional feedback loop is initiated by the Clock:Bmal1 dimer which involves E-box mediated transcription of orphan nuclear-receptor genes *Rev-Erb*α/β and *ROR*α/β (Preitner et al., 2002; Sato et al., 2004; Guillaumond et al., 2005). Rev-Erb has been implicated in normal period regulation (Cho et al., 2012), which competes with ROR proteins for Retinoic acid-related Orphan receptor Response Element (RORE) binding sites which are located within the promoter of *Bmal1*. ROR proteins are responsible for initiating *Bmal1* transcription, whereas Rev-Erb inhibits it (Preitner et al., 2002).

### Molecular Mechanisms Entraining and Modifying Central and Cellular Circadian Clocks

**FIGURE 1:**
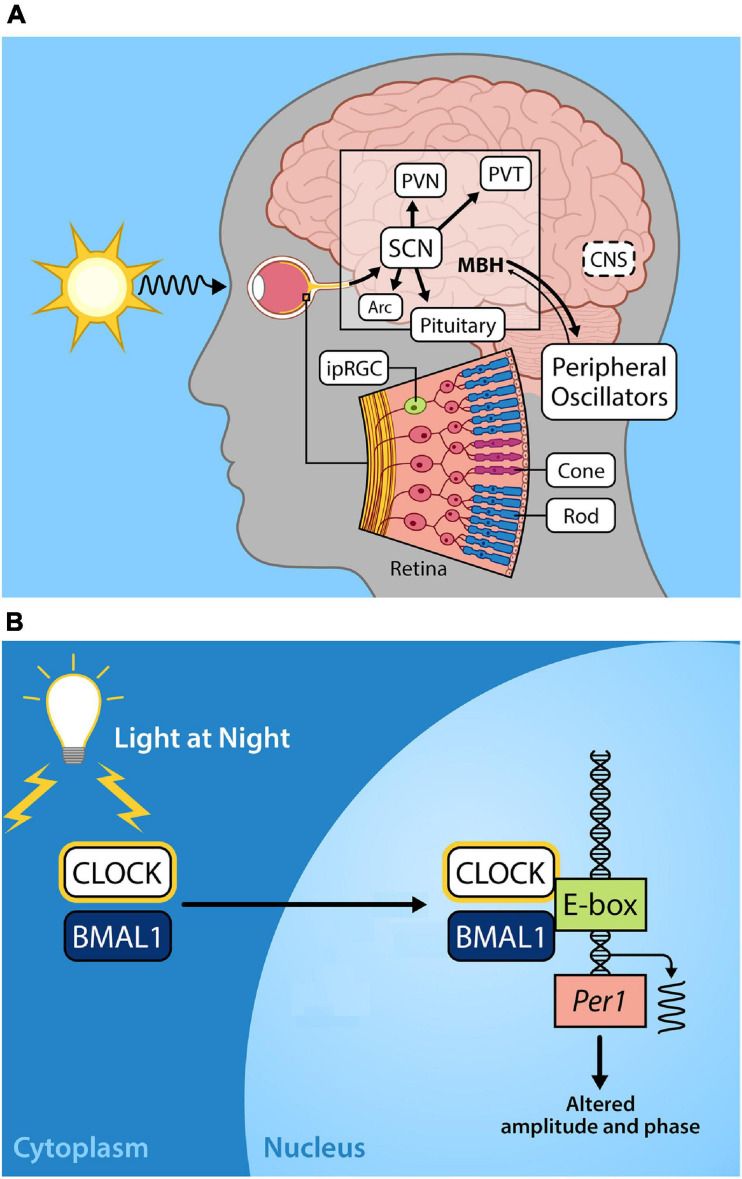
**(A)** Light entrains the circadian clock. The light-dark cycle is the strongest zeitgeber, involved in synchronizing intrinsic circadian oscillations in the brain and periphery. Light energy in the form of photons enter the retina, depolarizing intrinsically photosensitive retinal ganglion cells (ipRGCs) which relay photic information via the retinohypothalamic tract to the so-called master circadian pacemaker, the suprachiasmatic nucleus (SCN) in the anterior hypothalamus. Direct and indirect projections of the SCN entrain other regions including local nuclei within the mediobasal hypothalamus (MBH), the paraventricular nucleus (PVN), paraventricular nuclei of the thalamus (PVT), arcuate nucleus (Arc). Endocrine signals from the pituitary gland and the pineal (melatonin secretion) that are initiated by the SCN provide time of day information to entrain peripheral oscillators. **(B)** Exposure to light at night disrupts the molecular circadian clock. The molecular clock in the cells of central and peripheral tissues is comprised of transcriptional-translational feedback loops. The positive arm of the core circadian feedback loop involves the protein products of the genes circadian locomotor output cycles kaput (CLOCK) and brain and muscle arnt-like protein-1 (BMAL1), which heterodimerize in the cytoplasm, translocate to the nucleus, and bind to the E-box promotor regions of Period (Per) and Cryptochrome (Cry), driving their transcription. In the negative feedback arm of the core circadian feedback loop (not shown) Per/Cry protein products translocate to the nucleus to repress CLOCK/BMAL transcriptional activation, cycling with a period lasting approximately 24 h (please see Hurley et al., 2016; Rumanova et al., 2020) for further details on the molecular circadian clock). Exposure to light during the night disrupts Per1 expression resulting in altered circadian amplitude and phase.

Photoentrainment of the central circadian clock occurs primarily via light signals to intrinsically photosensitive retinal ganglion cells (ipRGCs) that depolarize and project through a monosynaptic neuronal pathway known as the retinohypothalamic tract (RHT) to the suprachiasmatic nucleus (SCN). The SCN serves as the master circadian clock in mammals and coordinates internal circadian synchronization; it sits at the top of a hierarchy of clocks of various tissue and cell types that display independent circadian gene expression patterns that are entrained by neural and humoral signals from the SCN (Tosini and Menaker, 1996; Balsalobre et al., 1998; Yamazaki et al., 2000). These non-image forming ipRGCs also function to regulate pineal melatonin secretion, the sleep/wake cycle, and pupillary constriction (Fu et al., 2005; Gamlin et al., 2007; Do and Yau, 2010) (Figure 1A).

Within the SCN, *Per* paralogs function as the state variable such that variations in the levels of proteins account for the phase of the clock (Kim et al., 2018). Acute exposure to light at any point during the dark period alters *Per1* expression in the core of the SCN (Shearman et al., 1997; Shigeyoshi et al., 1997) resulting in physiological and behavioral phase advances or delays. Exposure to light during the early dark phase results in increased Per2 expression in the shell of the SCN whereas light exposure late in the dark phase increases Per1 expression in the shell of the SCN, leading to a phase delay or phase advance in activity rhythms, respectively (Yan and Silver, 2004). This activation of *Per* paralogs occurs through CREB/MAPK signaling acting on cAMP-response elements (CRE) in *Per* promoters (Travnickova-Bendova et al., 2002). The functions of mammalian cellular clock proteins are additionally regulated through posttranslational modifications through various kinases, methylation, polyadenylation, histone modifications, and non-coding RNAs (Cardone et al., 2005; Mendoza-Viveros et al., 2017; Okamoto-Uchida et al., 2019). Phosphorylation was first discovered to be of major importance in vertebrate circadian clock regulation in the *tau* mutation in Syrian hamsters, where a lowered rate of CKI-dependent phosphorylation of Per2 occurred, resulting in shortened period of free running rhythms (Lowrey et al., 2000). Other post-translational regulators including Casein kinase II (CKII), PI3-kinase (PI3K), and c-Jun N-terminal kinases (JNKs) have also been implicated in modifying the cellular circadian clock, and may be critical for precise control of cellular circadian rhythms. The molecular details of precise regulation of the cellular circadian clock are outside the scope of this review; however, we note that stability, subcellular localization, transcriptional activity, and interactions among proteins and signaling pathways are also under circadian control (reviewed in Okamoto-Uchida et al., 2019).

## Sources of Circadian Disruption

Daily rhythms of natural bright days and dark nights have shaped the evolution of internal biological clocks almost since the emergence of life, from physiological pathways to behavior. During the past century, widespread adoption of electric lighting and shifts in daily activity in the form of night shift work and social jet lag schedules has drastically affected circadian organization. Despite the seemingly innocuous nature of prolonged and inconsistent exposure to light in the evening, phase shifts from shift work, or social/leisure activities, several aspects of behavior and physiology have been affected (Navara and Nelson, 2007).

### Environmental Lighting

The light-dark exposure patterns of humans have vastly changed over the past 100 years with the progression of civilization and industrialization, resulting in exposure to artificial light at night, or alternatively known as light pollution. Sources of illumination include street, architectural, and vehicle lighting, producing radiance from towns and cities via sky glow. Furthermore, light pollution introduces intensities and spectrums of light that differ significant from naturally occurring starlight and moonlight (Gaston et al., 2014). Approximately 5 lux of light exposure during the night is consistent with current light pollution levels in urban areas (Gaston et al., 2012) and sleeping environments (Obayashi et al., 2018). In comparison, the full moon is generally 0.05-0.2 lux of light at night (Kyba et al., 2017b). The light spectrum also varies depending on the light source used, ranging from high pressure sodium bulbs with broad bands of mainly yellow-orange wavelengths to high intensity discharge and light-emitting diodes (LEDs) producing narrow bandwidths with a strong peak of blue wavelengths in the spectrum, creating the appearance of whiter lighting (Falchi et al., 2011). This shift from incandescent bulbs to LEDs occurred during the early 21st century based on reduced energy consumption and cost from the more energy efficient bulbs. IpRGCs are most sensitive to short wavelength (i.e., blue/violet light; ∼459 nm) light (Wahl et al., 2019), and exposure to as little as 1.9 μW cm^–2^ of light at night suppresses nightly melatonin production in humans (Thapan et al., 2001). Suppression of melatonin induced by lighting exposure during the night has been replicated in other mammalian species (Klein and Weller, 1972; Rollag et al., 1980; Benshoff et al., 1987).

This is a concern given that a recent study using light pollution propagation software and satellite data reports that 83% of the world’s population reside under light-polluted skies (>μcd/m^2^), with that statistic jumping up to 99% in the United States and European populations (Falchi et al., 2016). Furthermore, light pollution levels have increased at a rate of 2.2% per year (Kyba et al., 2017a). Given the importance of light as a zeitgeber for humans and other species, nocturnal light exposure likely has serious consequences for health and wellbeing. Exposure to light during the dark phase can phase advance or delay the circadian clock (Schwartz et al., 2011), depending on the duration, timing, and intensity of the lighting source (Figure 1B). Low levels of artificial light at night (5 lux) disrupt circadian rhythmicity with a reduction in Per1 and Per2 expression in the SCN, concurrent with altered expression of *Bmal1, Per1, Per2*, and *Cry1* in the liver of mice (Fonken et al., 2013).

### Night Shift Work, Travel, and Social Jet Lag

Coinciding with the increase in light pollution as a result of industrialization, current society has deviated from “9 to 5” workdays; the majority of the working population is engaged in “non-standard” or irregular hours, encompassing on-call work, weekend work, compressed weeks, telework, split-shifts, or more classically thought of as night shift work (Costa et al., 2004). Indeed, 15–30% of adults are engaged in shift work according to American and European surveys (Boivin and Boudreau, 2014). Social jet lag, defined as the difference in lighting exposure and sleep time between work days and free days, is another form of circadian disruption that has become especially prevalent in adolescent and young adult populations (Valdez et al., 1996; Wittmann et al., 2006; Crowley et al., 2007). During social, travel jet lag, or shift work, desynchronization of central and peripheral oscillators occurs. Misalignments of circadian rhythms are responsible for “jet lag” syndrome characterized through fatigue, disturbed sleep rhythms, reduced alertness and ability to perform cognitive tasks, and headaches (Choy and Salbu, 2011).

## Molecular Clock’s Influence on Cardiovascular, Cerebrovascular, and Immune Parameters

Circadian regulation of cardiovascular and cerebrovascular physiology is well documented (Kostenko and Petrova, 2018; Thosar et al., 2018), and includes heart rate and heart rate variability (Massin et al., 2000), sympathetic tone (Panza et al., 1991), blood pressure (Coca, 1994), and cerebral blood flow (Conroy et al., 2005; Hodkinson et al., 2014). Coordinated circadian patterns of these key physiological measures are crucial for meeting the increased physical demands of the active part of the day and the reduced demands of sleep. Aberrations in temporal rhythms can both directly and indirectly enhance cardiovascular and cerebrovascular disease (Bøggild and Knutsson, 1999), by enhancing sympathetic drive (Morris et al., 2012) increasing obesity (Shi et al., 2013), inducing insulin resistance and metabolic disorders (Marcheva et al., 2013), promoting premature aging (Brown et al., 2011), and inflammatory pathologies (Savvidis and Koutsilieris, 2012; Salavaty, 2015). These physiological perturbations can interact with cardiovascular phenotypes and prothrombotic states to play a significant role in stroke recurrence, functional outcome (Arboix, 2015), and outcome with pharmacological intervention with tissue plasminogen activator (tPA) (Seet et al., 2014). Cardiovascular tissue displays robust circadian oscillations in cells including vascular smooth muscle, fibroblasts, cardiomyocytes, and cardiac progenitor-like cells, all of which regulate physiological functions including endothelial function, blood pressure, and heart rate (Paschos and FitzGerald, 2010).

At the cellular level, the core clock genes, including *Bmal1, Clock, Per, Cry*, and *Rev-Erb*, play an important and vital role in maintaining physiological homeostasis. In addition to studies evaluating the role of time of day, genetic approaches can be used in basic science models to assess the role of specific genes within the molecular circadian clock. Notably, *Bmal1, Clock, 1/2 Per* double knock-out, and *Cry1/2* double knock-out mice are completely arrhythmic (van der Horst et al., 1999; Bunger et al., 2000; Bae et al., 2001; DeBruyne et al., 2007), whereas single knockouts of *Cry* and *Per* have altered periods (van der Horst et al., 1999; Bae et al., 2001). Please see Cermakian et al. (2001) for a more extensive review of the circadian phenotypes of genetically altered models.

The next section will discuss how aberrations to circadian expression rhythms from environmental and genetic factors will alter cardiovascular, cerebrovascular, and immune parameters that increase stroke risk and contribute to the pathogenesis, damage, and outcome of cerebral ischemia (Brown et al., 2009; Ramsey et al., 2020).

### Coagulation, Hemostasis, and Fibrinolysis

In response to vascular injury, platelets are activated upon coming in contact with subendothelial matrix proteins; platelet activation is responsible for clot formation in the procoagulant pathway, while several inhibitors function to inhibit clot propagation and avoid thrombus propagation (Palta et al., 2014). Several pro-inflammatory mediators including chemokines, adhesion molecules, vasoactive mediators, growth factors, and surface ligands are released upon platelet activation (Morrell et al., 2007). Indeed, this system relies on the intricacy of two systems in order to maintain fluidity and circulation of blood. In the context of cerebral ischemia, platelets play a crucial role in the pathogenesis of this condition; time of day alterations in factors involved in hemostasis and fibrinolysis contribute to a hyperfibrinolytic state, in turn increasing risk for stroke during morning time points.

In a study evaluating *in vitro* platelet aggregation in response to adenosine diphosphate (ADP) and adrenalin, platelet adhesiveness was measured using blood samples from healthy subjects (5 males and 5 females) over six timepoints (0800, 1200, 1600, 2000, 0000, and 0400 h); prothrombin peaked at 1600 h, suggesting a transient morning hypercoagulable state (Haus et al., 1990). Increased platelet activation along with fibrinogen, plasminogen-activator inhibitor 1 (PAI-1), plasmin-alpha-2-antiplasmin (PAP), and thrombin-antithrombin (TAT) complexes has been observed in healthy human volunteers (20-50 years old) during the morning (0800 h), with increasing concentrations of endogenous tPA, d-dimers, and PT prolongation in the afternoon (Budkowska et al., 2019). Clotting times were significantly reduced during the active period (night) in nocturnal rats, with increased prothrombin (II), factor VII, and factor X during the inactive period (day) (Soulban and Labrecque, 1989).

Coagulation and fibrinolysis has been further investigated in *Clock* mutant and *Cry1/2* knockout (arrhythmic) mice; euglobulin clot lysis time was reduced in *Clock* mutants and significantly increased in *Cry1/2*-deficient mice without time-of-day differences (0900 and 2100 h). Further characterization showed reduced PAI-1 fluctuation in *Clock* mutant mice while *Cry1/2*-deficient mice held similar levels at both 0900 and 2100 h (Ohkura et al., 2006). Deficiencies in *Bmal1*, showed hypercoagulable states, increased arterial and venous thrombogenicity, that lead to progressive dysfunction in endothelial cells and subsequent increased platelets and factor VII with age (Hemmeryckx et al., 2019). In common with these findings, vascular occlusion time was increased in this mutant mouse (Westgate et al., 2008). *Bmal* knockout (arrhythmic) mice display progressing prothrombotic state characterized by reduced prothrombin times, increased platelet count, a reduction in nitric oxide/thrombomodulin expression from endothelial cells (Hemmeryckx et al., 2011). *Clock* is also involved in the regulation of hemostasis. Mutations in *Clock* increased the total and active PAI-1 levels along with reduced tPA in plasma (Westgate et al., 2008). Taken together, these results suggest that core clock genes are involved in regulating expression of key components in hemostasis and the fibrinolytic system, leading to an increased risk for the development of prothrombotic phenotypes and thus, an increased risk for cerebrovascular events.

### Vascular Function and the Blood-Brain Barrier

The vascular system, specifically, the cerebrovasculature, has been implicated as an important role in brain development and regulation of homeostasis. Endothelial cells comprise the inner monolayer of blood vessels that forms a barrier between the artery wall and circulating blood is responsible for regulating vascular tone, regulation of hemostatic properties on the vascular surface, and determining blood-tissue/brain permeability (Paschos and FitzGerald, 2010). The blood-brain barrier (BBB), is a tightly regulated system composed of transporters including permeability-glycoprotein (Pgp) and ATP-binding cassette sub-family B member 1 (ABCB1). Movement across the BBB occurs via transmembrane efflux pumps (Abbott et al., 2010) that transport molecules through endocytosis or through pores, carrier-mediated transport systems, or through direct permeation through the plasma membrane via lipophilic molecules (Banks and Kastin, 1992). These efflux pumps are regulated by ATP-binding cassette (ABC) transporters or paracellular aqueous diffusion which are inhibited by tight junctions.

The BBB plays an integral role in the neuronal damage that evolves from disrupted blood flow. As a result of impaired blood supply, essential nutrients such as glucose and oxygen fail to adequately reach the ischemic core and surrounding region known as the penumbra. Endothelial ion transporters, including the Na-H and Na-K-Cl cotransporter, become dysregulated upon neuronal injury, resulting in an imbalance of ionic gradients and cytotoxic edema that contribute to secondary neuronal damage (Pinheiro et al., 2016). Additionally, increased paracellular permeability (Keaney and Campbell, 2015), and increased immune cell trafficking, including infiltrating leukocytes, can exacerbate inflammatory responses, leading to increased neuronal damage and injury (Huang et al., 2006). Furthermore, disruptions to the BBB following thrombolytic intervention can be an early predictor for hemorrhagic transformation (Kastrup et al., 2008).

Evidence of an endogenous circadian rhythm in blood brain barrier permeability has been characterized in Drosophila where rhodamine B and daunorubicin concentrations were significantly increased in the brain during the rest phase (ZT 12) compared to their active phase through increased efflux activity via pgp-like transporters during the day (Zhang et al., 2018). This oscillation was abolished in *Per* deficient flies (altered period) (Zhang et al., 2018). Further, several molecules, including tumor necrosis factor alpha (TNFα), leptin, β-amyloid, delta-sleep inducing peptide (DSIP), and prostaglandin D2 (PGD2) display changes of rhythmic entry into murine CNS (Cuddapah et al., 2019). Other aspects of the blood brain barrier including integrity are also under circadian control. *Bmal1* deficient mice display hyperpermeability and downregulation of platelet-derived growth factor receptor β (PDGFRβ), suggesting a significant role for *Bmal1* in pericyte regulation for blood-brain barrier maintenance (Nakazato et al., 2017).

Secondly, vascular function and tone also demonstrate robust core clock rhythms, best characterized through blood pressure peaks which are observed prior to the onset of the active cycle (light phase in diurnal species) followed by mid-morning peaks and decreases during the evening (inactive phase) (Millar-Craig et al., 1978). Additionally, a time-of-day dependent vasoreactivity exists (Durgan et al., 2017), further supporting the notion of circadian regulation of the cardiovascular system. *Bmal1* knockout mice display lower blood pressure and abolished rhythmicity (Curtis et al., 2007), whereas *Per1* has been implicated in aldosterone (Richards et al., 2013) and sodium regulation (Stow et al., 2012), both of which play a role in blood pressure maintenance. Selective deletion of Bmal1 in cardiomyocytes results in decreased heart rate from altered Na^+^ and K^+^ channels (Schroder et al., 2013), while deletion in smooth muscle cells advances systolic and diastolic blood pressure (Xie et al., 2015). Furthermore, *Bmal1* deficient mice exhibit acute vascular dysfunction, vascular remodeling, and injury (Anea et al., 2009).

Nitric oxide (NO) and redox signaling involved in vascular contraction is another process that displays robust circadian oscillations involved in the vasoconstriction process. In the context of cerebral ischemia, NO synthases have both neuroprotective and damaging effects during brain hypoxia. Inducible NOS (NOS2) and neuronal NOS (NOS1) are involved in neurotoxicity, leading to excitotoxicity cascades, inflammation, apoptosis, and deterioration to the primary brain injury through the release of free radicals and production of nitrates, which directly damage mitochondrial enzymes and genetic material (Sims and Anderson, 2002; Chen et al., 2017). Endothelial NOS (NOS3) has a neuroprotective effect through regulation of cerebral blood flow, preventing neuronal injury, and inhibiting platelet and leukocyte adhesion (Liu et al., 2014). A study examining time of day variation in contractility response in mesenteric arteries isolated from Wistar rats reported increased NOS3 expression during the active period, which coincided with reduced vasoconstriction in response to phenylephrine and increased vasodilation response to acetylcholine during the active period (Rodrigo and Denniff, 2016). Further, NOS activity and cytosolic protein content was evaluated in Sprague-Dawley rats at 0300, 0900, 1500, and 2100 h in various regions of the brain including cerebellum, brainstem, hypothalamus, and hippocampus and reported that a significant upregulation during the dark (active) period (Ayers et al., 1996). Both endothelial nitric oxide synthase and NADPH oxidases (Nox), two factors involved in producing vasodilators (EDRF, EDHF, and PGI2) exhibit circadian variations in vasculature (Denniff et al., 2014). In addition to this time of day variation, disruption of circadian rhythms from exposure to dim light at night (dLAN; 1-2 lux) resulted in increased NOS3 protein expression in the arteries after 2 weeks of exposure in male Wistar rats (Molcan et al., 2019). Furthermore, NOS3 knockout mice display increased infarct sizes compared to wild type mice post middle cerebral artery occlusion (MCAO) (Huang et al., 1996). As mentioned above, NOS has significant neurotoxic impacts, and acts as a neuroprotective agent through mediation of vasodilation, plays a role in platelet aggregation inhibition, and contributes to endothelial leukocyte adhesion (Huang, 2007), and the studies above provide evidence and further support that disruptions to circadian rhythms support and increase neurotoxic effects and dampened neuroprotective aspects of NOS. In conclusion, several aspects of the vascular system are critically involved in acute injury and recovery from cerebral ischemia and are tightly regulated by core clock genes. Time of day alterations in function exist, suggesting a potential avenue for targeted therapeutic intervention. Thus, the BBB has reduced trafficking during the active phase compared to other time points during the day, along with an increased morning surge in blood pressure contributing to a heightened prothrombotic state, increasing risk for ischemia. Additionally, morning time points and studies evaluating circadian disruption have increased NOS, that can contribute toward neurotoxicity and secondary ischemic damage. Disruptions circadian rhythmicity in core clock gene expression patterns has vast implications contributing to increased stroke risk and impaired recovery.

### Immune System

Components of the immune system are critical in pathophysiology of cerebral ischemia and are also regulated by the circadian clock. Acute cerebral hypoxia caused by thrombosis during ischemia triggers inflammatory cascades, and immune activation can cause secondary damage within the penumbra of the ischemic infarct (Kamel and Iadecola, 2012). Molecular clocks have been discovered in immune cells including macrophages/monocytes, T cells, NK cells, dendritic cells, and B cells (Ella et al., 2016), along with a circadian regulation in recruitment and infiltration of immune cells including neutrophils, monocytes/macrophages, and T cells, which infiltrate the injury site after the integrity of the BBB is compromised (Scheiermann et al., 2013). Rhythmic daily oscillations in both circulating innate and adaptive immune cells, and in cytokine and chemokines occur in the healthy brain (Labrecque and Cermakian, 2015), and circadian clock disruptions dysregulate immune response (Comas et al., 2017) and alters circulating proinflammatory cytokines, complement factors, and oxidative stress (Shivshankar et al., 2020).

In addition to the highly organized and regulated immune system, there are specific immune cell populations that exist in the CNS that are vital in maintaining normal brain homeostasis and function. Glial cells including microglia and astrocytes are non-neuronal populations in the central nervous system that function to maintain homeostasis, provide support, and protection, which are both involved in the innate and adaptive immune system (Herculano-Houzel, 2014; Zuchero and Barres, 2015). Inflammation is a key component and contributor in the pathophysiology of ischemia, existing in every stage of the ischemic cascade (Iadecola and Anrather, 2011). Upon neuronal and cell injury from ischemic infarction, glial populations function as the primary proponents in the early peri-infarct environment, however, have been implicated in both beneficial and a detrimental impact on the ischemic core (Xu et al., 2020).

Microglia are a glial cell that plays an active role in immune surveillance in the central nervous system and are heavily involved in the initiation of the innate and adaptive immune response (van Rossum and Hanisch, 2004). This cell population accounts for up to 15% of all cells found within the brain (Lawson et al., 1992). This population plays a crucial role in triggering innate immune response by releasing pro and anti-inflammatory cytokines, chemokines, nitric oxide, prostaglandins, growth factors, and superoxide species that can modulate secondary injury and recovery (Loane and Byrnes, 2010). Microglia also recruit leukocytes, myeloid dendritic cells, monocytes/macrophages, and neutrophils to the site of ischemic damage during the earlier stages of stroke (Iadecola and Anrather, 2011; Jian et al., 2019). This activation process has a central role in initiating a neuroinflammatory response post stroke which can cause pathological progression of damage in the ischemic penumbra, however, aspects of the neuroinflammatory response are also critical for tissue repair (Yenari et al., 2010). Microglia are under circadian clock control, with robust hippocampal TNFα, IL1β, and IL6 expression, peaking during the light or inactive phase in male Sprague-Dawley rats, with increased cytokine expression in response to an immune stimulation with lipopolysaccharide (LPS) during the light/inactive phase (Fonken et al., 2015). Microglial activation, and interleukin expression specifically, is regulated by *Bmal1* expression (Takano et al., 2009), along with a circadian variation in markers involved in oxidation, NADPH oxidase 2 (Nox2), and inflammatory TNFα, IL1β and IL-6 in microglia increased during the light or inactive phase in nocturnal mice. Conversely, glutathione reductase (Gsr), heme oxygenase 1 (Hmox1), glucose transporter member 5 (Glut5) and lipoprotein lipase (Lpl) were increased during the dark, or active period, suggesting that microglia have increased nutrient uptake during the active period. Wang et al. continued using *Bmal1* knockout mice and reported altered clock gene expression (Cry2 and Per2) along with decreased Nrd1, Dbp, Il1 β, and Nox2 expression and increased Gsr and Lpl expression, indicating that *Bmal1* regulates immune response and cellular metabolism in microglia (Wang et al., 2020). Other circadian clock proteins, such as Rev-erbα have also been implicated as a mediator of microglial activation and neuroinflammation, where the authors observed a time of day difference in microglial immunoreactivity in the hippocampus (Griffin et al., 2019). Further examination with Rev-erbα knockout mice, indicated increased basal nuclear factor κB (NFκB) and enhanced hippocampal neuroinflammatory reactivity after inflammatory challenge (Griffin et al., 2019) providing further evidence of the role and importance of circadian clock regulation on immune function. These results also highlight the detrimental effects that result from altered circadian rhythms, and the downstream dysregulation of this highly conserved system.

Astrocytes are another specialized glial cell population integrated into both the vascular and neuronal system; and are critical for maintenance and survival of neurons during normal brain function (Stubblefield and Lechleiter, 2019). In the context of cerebral ischemia, astrocytes are especially important in regulation and maintenance of glutamate during acute injury, which causes excitotoxicity and perturbates calcium influx resulting in cell death (Brancaccio et al., 2017). Processes under astrocyte control including glutamatergic signaling (Beaulé et al., 2009) are regulated through the molecular clock, and in addition, extracellular glutamate displays rhythmicity, peaking during the mid-late day (inactive period) in mice. Disruptions to core clock genes including *Per2, Clock*, and NPAS2 display significant reductions in glutamate uptake, mRNA expression of Glast, an astrocyte-specific glutamate transporter, and protein expression (Sofroniew, 2015). Astrocytes are also involved in the recruitment of inflammatory cells to the site of injury, through the formation of a glial scar termed reactive astrogliosis which involves activation and proliferation of astrocytes to limit extending damage (Lananna et al., 2018). Astrocytic activation is regulated by *Bmal1*, shown through activation and increased inflammatory gene expression using an astrocyte specific *Bmal1* knockout (Ali et al., 2020). Other studies using *Bmal1* knockouts in astrocyte cultures have reported reduced actin-binding protein cortactin and impaired actin stress fiber formation (Ali et al., 2020).

Other immune cell populations that are involved in regulating the progression of ischemic injury are neutrophils, macrophages, and T-cells (Jian et al., 2019). As mentioned, the immune system is a tightly regulated system which is important to maintain health and survival. Appropriate inflammatory balance is especially important during ischemic injury, where there is major neuronal death and without proper neuroprotective measures, progressing injury can have detrimental effects on functional outcome and survival. Neutrophils are among the first immune cell populations to respond to ischemic injury; they are important for their phagocytotic properties to remove necrotic debris, but can result in neuroedema, disruption to the blood brain barrier, and collateral tissue damage (Jickling et al., 2015). Neutrophils have been well characterized to display robust oscillating rhythms in blood (Casanova-Acebes et al., 2013), variations in *Bmal1* expression (Gibbs et al., 2014), and time-of-day regulated infiltration into bone marrow, lung, liver, and spleen tissue (Casanova-Acebes et al., 2018). Macrophages and monocytes are other immune cells in the mononuclear phagocyte system that have robust cell-autonomous rhythms (Scheiermann et al., 2013). Clock genes such as Rev-erb repress distal enhancers resulting in the repression of macrophages (Lam et al., 2013), whereas *Bmal1* which controls rhythmic trafficking of inflammatory monocytes to sites of inflammation (Nguyen et al., 2013). In the context of stroke, macrophages and monocytes function similar to neutrophils and are especially important for their phagocytotic abilities during ischemia. T-cells are one of the major white blood cell types involved in the adaptive immune system and migrate to the site of ischemic injury after the initial inflammatory cascade within 24 h (Jian et al., 2019) which is dependent on adhesion molecule expression in the endothelium (Arumugam et al., 2005). This infiltration is important, due to the high mortality rate associated with infection during the acute phase of stroke recovery (Shi et al., 2018). Helper T cells (TH1) can play a neuroprotective role during recovery through anti-inflammatory cytokines (IL-4, IL-5, IL-10, IL-13), however, cytotoxic T cells can also contribute toward secondary injury from cytotoxic granules (Arumugam et al., 2005). Circadian variations in T cell proliferation in the lymph nodes exist, where T cell proliferation was significantly greater during the dark or active period in mice compared to the light, which was abolished in *Clock* mutant mice (Marie-Pierre Hardy et al., 2021). Based on the crucial role that timing plays in stroke intervention, we conclude that circulating immune factors and immune response is most active during the morning. Upon neuronal injury, heightened immune activation is observed during the morning increasing the risk for neurocytoxicity and secondary damage compared to the evening. In addition, disruptions to circadian rhythms and core clock gene function through exposure to light at night disrupts immune function and increases neuroinflammation (Walker et al., 2020), that has been corroborated in an ischemic murine model in which 24 h of exposure to dLAN post stroke increased pro-inflammatory response (TNF-α, IL-6, and IL-1) in the ipsilateral cortex that can further amplify secondary damage of the infarct (Weil et al., 2020). Disruptions to circadian rhythms can lead to a prothrombotic state, in which patients during the morning time point are especially at risk for poor outcome, further highlighting the importance of time of day when considering therapeutic strategies.

## Clinical Studies, Disruptions to Circadian Rhythms, and Stroke

During the late 20th century hospital analyses began to investigate the correlation between circadian variations in ischemic onset and outcome, highlighting the increased morning frequency of this cerebrovascular event. A meta-analysis of 31 publications (11,816 strokes) revealed a 49% increase of occurrence between 0600 and 1200 h in all types of strokes, and 79% increase over normalized risk compared to the other 18 h of the day (Elliott, 1998), with the lowest incidence of stroke reported between 0000 and 0600 h (Elliott, 1998). Additionally, mortality rates for strokes that occur during the morning were significantly higher, even when adjusted for sex, severity, and age (Turin et al., 2012). Analysis of stroke bank data revealed a significant increase in the number of strokes in awake patients from 1000 to 1200 h, compared to any other 2-h interval throughout the day (Marler et al., 1989). Another study of 1223 patients reported increased frequency of acute ischemia between 0600 and 1200 h; lacunar, thrombotic, and embolic strokes displayed increased frequency between 1801 and 0000 h (Lago et al., 1998). Ischemic stroke patients who experience “Wake-up Strokes,” characterized by patients who were asleep for greater than 3 h, with noted stroke symptoms upon waking between 0100 and 1100 h had increased diastolic blood pressure, a longer hospital stay. Further, this study indicated that patients were also at an increased risk for in-hospital mortality (Denny et al., 2014). In addition to an effect of time of day on risk, disrupted circadian rhythms also increase risk. Rotating shift work, which disrupts circadian rhythms, has also been associated with a 4% increased risk for ischemic stroke for every 5 years, with an increased risk of stroke in women with longer durations of shift work (≤ 15 years) (Brown et al., 2009).

Together, the literature supports the notion that a time of day variation in stroke outcome exists, and that patients who experience stroke-like symptoms during the morning, or onset of the active period, have an increased risk for poor outcome. The data also suggest that it likewise could be possible to optimize therapeutic strategies based on the biological time that the onset of stroke occurs to improve survival rates. Time of day and circadian rhythms are both important variables in ischemia, and few clinical studies have considered these variables as biological factors. In this review we highlight the existence of circadian variations in ischemia and efficacy of thrombolytic intervention for clinicians, physicians, and pharmacists with the aim that time of day be recorded and reported as a biological variable in future clinical studies (Nelson and DeVries, 2017).

Other aspects of temporal organization influence and alter stroke recovery. For example, exposure to artificial lighting from hospital recovery units is a major issue (Korompeli et al., 2017). Indeed, clinicians need to monitor and treat patients 24 h a day, however, patients may be especially vulnerable to the influence of light at night due to their compromised state; frequent aspects of intensive hospital care units include frequent waking for vitals and procedures, and noise from equipment and staff (West et al., 2017). Few clinical studies to our knowledge have directly evaluated the role of dim nighttime lighting compared to dark nights on functional recovery and outcome in stroke, however, literature focused on normalizing naturalistic lighting during recovery have shown significant improvements (Wright et al., 2013). In the context of disrupted circadian rhythms, the use of naturalistic lighting optimizes light exposure similar to solar time, allowing for resynchronization of the internal biological clock to the natural light dark cycle (West et al., 2019). Using 24 h naturalistic lighting in rehabilitation units post-stroke found that patients experienced improved sleep with decreased disturbances, improved cognitive function, and endocrine function (Wang and Chen, 2020). Other studies using artificial sunlight exposure therapy during the day, with increasing morning blue-spectral illuminance peaking in the afternoon to imitate daylight for a minimum of 14 days of exposure in 4 weeks post stroke reported improved daily function through the Bartel Index and depressive test scores (Durgan et al., 2017). Thus, normalization through re-entrainment of circadian rhythms to light dark cycles post cerebrovascular injury through the use of controlled lighting in a hospital setting is a non-invasive intervention that can potentiate improved recovery rates.

## Time of Day Alterations on Ischemic Stroke Outcome (Basic Science)

As highlighted above, stroke incidence has a distinct time of day frequency, where studies identified a morning increase in frequency and mortality compared to any other time point in clinical studies (Marler et al., 1989). In addition to these time of day variations, basic science research has identified a circadian aspect in stroke severity, infarct volume, and functional outcome in rodents (Weil et al., 2009; Beker et al., 2018; Ramsey et al., 2020). In a controlled study, variation in ischemia/reperfusion tolerance across time of day has been previously explored, showing an increase in infarct volume and fibrosis at the onset of activity (ZT12) compared to the beginning of the inactive period (ZT0) in WT mice (Durgan et al., 2010). In addition, genetic ablation of the molecular clock in cardiomyocytes abolished this diurnal variation (Durgan et al., 2011). Global cerebral ischemia from cardiac arrest during the light (inactive) phase impaired survival and exacerbated outcome characterized by increased microglial activation, degenerating neurons, and proinflammatory cytokines in the hippocampus compared to dark (active) phase in C3H mice (Weil et al., 2009). Studies into the molecular mechanisms underlying the time of day differences post ischemia revealed that ischemic/reperfusion injury at ZT 18 had reduced infarct volume, edema, neurological deficits, apoptotic death, and improved neuronal survival compared to other time points (ZT0, ZT6, ZT12, and ZT18), coinciding with a decrease in PRAS40 and increased expression of *Bmal1, Per1, Clock, AKT, Erk-1/2, mTOR, S6*, and *BAD* (Beker et al., 2018).

### Disruptions to Circadian Rhythms Influence on Ischemic Stroke (Basic Science)

Disruptions to circadian rhythms prior to ischemic events leads to a prothrombotic state resulting in a heightened predisposition for enhanced stroke damage and poor outcome. Chronic circadian disruption through phase advances increased infarct volume in mice that received an MCAO (Ramsey et al., 2020). Another recent study reported that chronic disruption of circadian rhythms by a 12-h phase advance every 5 days for 7 weeks prior to MCAO increased infarct size in male and female Sprague Dawley rats. Further, male rats had increased mortality, while surviving females displayed a significant decrease in serum IGF-1 and nearly a doubling of infarct volume and sensorimotor deficits (Earnest et al., 2016).

In addition to disruption of circadian rhythms by repeated phase shifts (jet lag), foundational science research has examined the role of circadian disruption by light at night in stroke outcome. Studies evaluating the impact of ecologically relevant levels of dLAN (5 lux) have reported that stroke lesion size was significantly larger in dLAN mice compared to mice housed in dark nights after 24 h (Weil et al., 2020). Furthermore, this study reported that mice exposed to three days of dLAN post MCAO increased TNF-α, IL-6, and IL-1 in the ipsilateral hemisphere of the brain, and displayed increased anxiety-like behavior compared to control mice (Weil et al., 2020). Another study evaluating the effect of dLAN reports that as few as four days is sufficient to induce neuroinflammation in female mice (Walker et al., 2020) which can contribute toward neurotoxicity, increased neuronal damage within the penumbra, and delayed recovery post ischemia.

### Disruptions to Core Clock Genes (Basic Science)

Disruptions to core circadian clock proteins, specifically *Bmal1*, has also resulted in increased reactive oxygen species, which is critical during mediation/exacerbation of damage during reperfusion in ischemia (Peek et al., 2017). Meanwhile, overexpression of *Bmal1* and *Clock* may activate HIF-1α, involved in the regulation of innate neuroprotection (Lembach et al., 2018). Additionally, there is a difference between biological outcomes when comparing males and females post ischemic injury; the infarct core and both microglial activation and astrogliosis were significantly increased in females compared to males; furthermore, this sex difference persisted in female *Bmal1* knockout mice that displayed increased core infarct size compared to wild type females (Wiebking et al., 2013). *Per1* knockout mice increased neuronal cell death post ischemia compared to wild-type control mice. Importantly, ischemic injury during time points coincident with low *Per1* expression were more susceptible to apoptosis compared to high Per1 expression (Jickling et al., 2014). Disruptions to core clock gene expression, which can occur from exposure to light at night or repeated phase shifts resulting from social jet lag can result in significant disruption in the tissue recovery process, which in the context stroke, could result in increased ischemic damage and could be detrimental to patient outcome.

## tPA and Circadian Rhythms, and Factors Involved in Hemorrhagic Transformation

Blood-brain barrier dysfunction is a well-characterized occurrence post ischemia; however, this component also contributes to hemorrhagic transformation and increased mortality post thrombolytic intervention (Lo et al., 2004). Hemorrhagic transformation occurs through a disruption in tight junction proteins, and investigations into this underlying mechanism found that dysregulated extracellular proteolysis in the vascular matrix after tPA contributes to hemorrhagic transformation (Chandler et al., 1990; Kurnik, 1995).

Endogenous active tPA is inversely proportional with PAI-1 levels, peaking during the evening (Kastrup et al., 2008). A study evaluating blood-brain barrier opening shortly after cerebral ischemic onset correlated with an increased risk for intracerebral hemorrhage following exogenous tPA thrombolysis (Yang and Rosenberg, 2015). Furthermore, tPA intervention increased brain matrix metalloproteinase (MMP-9) (Yang et al., 2016), as well as phosphorylation of connexin43, a gap junction protein contributing to blood brain barrier permeability and hemorrhagic transformation (Tang and Han, 2013).

PAI-1, a serine protease inhibitor and another factor involved in the pathogenesis of stroke is produced primarily by the endothelium which directly acts as an active inhibitor of tPA and urokinase (uPA), a serine protease which is responsible for cleaving plasminogen to plasmin (Lakhan et al., 2013). Plasminogen regulates proteolytic and complement cascade (Barthel et al., 2012) which can contribute to thrombolysis or mediates degradation in conjunction with matrix metalloproteinases (Oishi, 2009). PAI-1 displays circadian rhythmicity, peaking during the morning (or the onset of the active period), in turn contributing to morning hypofibrinolysis (Schoenhard et al., 2003); elevated endogenous PAI-1 has been suggested to play a role in the morning peak of cardiovascular events (Scheer and Shea, 2014). Further *in vitro* work reported that the human PAI-1 promoter region in transfected endothelial cells is differentially activated by *Bmal1* and *Bmal2* (Somanath et al., 2011). *In vivo* work with *Bmal1* knockout mice showed shorter cessation of tail bleeding, occlusion time post injury, increased plasma fibrinogen, and cessation of rhythmicity for PAI-1 (Sumii and Lo, 2002).

Matrix metalloproteinases (MMPs) involved in vascular matrix remodeling, specifically, MMP-2 and MMP-9 are upregulated after tPA administration in focal cerebral ischemia (Wang and Chen, 2020); MMP-9 concentrations were reduced in tPA knock-out mice (Rosenberg, 2002). Furthermore, neuroedema was reduced after intracerebral hemorrhage through MMP inhibitor administration (Wang et al., 2003). Lipoprotein receptor-related protein (LRP) also known as LDLR, expressed in neurons, astrocytes, and endothelial cells in the brain functions as a receptor for tPA, mediating MMP upregulation, which in turn can contribute to hemorrhagic transformation from degradation of the neurovascular matrix (Lee et al., 2012). LDLR/LRP expression is under circadian control through *Clock/Bmal1* (Cappellari et al., 2014). Thus, LDLR/LRP may serve as a potential intervention point for time-of-day differences during ischemic onset and tPA intervention that could potentiate intracerebral hemorrhage.

### Clinical Studies and Time of Day Alterations in tPA Efficacy

**FIGURE 2:**
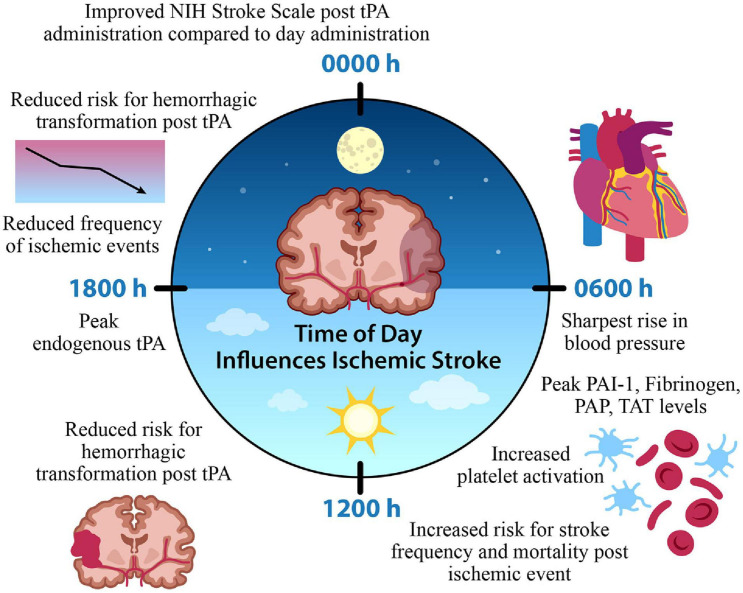
Time of Day Influences Ischemic Stroke. A time of day variation exists in the onset and frequency of stroke, factors contributing to cerebral ischemia, and the efficacy of thrombolytic medication administration. A morning surge of blood pressure, fibrinolytic agents, and platelet activation coincide with increased stroke onset. In contrast, the frequency of ischemic events is reduced during the evening, along with a reduced risk for hemorrhagic transformation and improved function with tPA intervention. This suggests a potential chronopharmacological therapeutic target for optimized treatment intervention that could improve patient outcome based on the time of ischemic event. Tissue plasminogen activator (tPA) plasminogen activator inhibitor-1 (PAI-1), plasmin-alpha-2-antiplasmin complex (PAP), thrombin-antithrombin complex (TAT).

In the context of clinical studies, a small population study of 476 non-lacunar strokes treated with an IV infusion of tPA was interested in identifying possible circadian variation with thrombolytic intervention; patients who received IV thrombolysis at morning (0600–1200) and afternoon (1200–1800 h) time points exhibited less improvement on the NIH Stroke Scale compared to other time points. Furthermore, hemorrhagic transformation was also lower in frequency for patients who started IV thrombolysis between 1200 and 1800 h compared to those who received treatment between 0000 and 0600 h (Marler et al., 1989). Together, evidence supports the role of time of day alterations in the efficacy of tPA and hemorrhagic transformation, as a potential negative consequence of tPA administration. Patients who have an ischemic stroke in the morning, or onset of their active period, are not only at a greater risk for poor outcome based on the hyperfibrinolytic morning state, but additionally, response to thrombolytic intervention is less efficacious during the morning time points. Alongside this, the time of day variation in stroke onset exists, where there’s a significant increase in morning onset strokes (Marler et al., 1989). This further emphasizes the potential avenue for a significant improvement in a large proportion of stroke patient outcome where targeted thrombolytic intervention is modified based on time of onset (Figure 2).

## Conclusion

Stroke requires a rapid diagnosis and quick intervention because of the limited time for thrombolytic intervention, highlighting the importance of timing for this disease. Biological timing and clock gene expression play important roles in the cerebrovascular and cardiovascular systems. Basic foundational research has clearly demonstrated time of day variation in function, and the detrimental consequences of disruptions to circadian rhythms and their impact on contributing to a prothrombotic state. In addition to the emphasized morning circadian distribution pattern of stroke frequency, clinical studies support improved functional outcome and recovery in patients who receive thrombolytic therapy during the evening; the morning hypofibrinolytic and hypercoagulable state, in combination with increased PAI-1 levels, may contribute to less effective tPA treatment in the morning. Furthermore, patients have significantly lower neurological improvement rates when tPA is administered in the morning compared to the evening, as well as increased hemorrhagic transformation post thrombolytic intervention during morning time points. Thus, optimizing thrombolytic treatment based on time of day onset and the reduction of exposure to exogenous environmental circadian disruptors, such as exposure to lighting at night, has the potential to reduce secondary damage during stroke recovery and improve functional outcome. The intricate relationship among circadian rhythms, disruptions, and the stroke outcome, emphasizes the importance of chronopharmacological research.

## Author Contributions

JL, JW, AD, and RN designed, wrote, and edited the manuscript. JL designed the figures. All authors have read and agreed to the published version of the manuscript.

## Conflict of Interest

The authors declare that the research was conducted in the absence of any commercial or financial relationships that could be construed as a potential conflict of interest.
